# Kinematic and kinetic assessment of upper limb movements in patients with writer's cramp

**DOI:** 10.1186/s12984-016-0122-0

**Published:** 2016-02-18

**Authors:** Mehdi Delrobaei, Fariborz Rahimi, Mallory E. Jackman, S. Farokh Atashzar, Mahya Shahbazi, Rajni Patel, Mandar Jog

**Affiliations:** K. N. Toosi University of Technology, Faculty of Electrical and Computer Engineering, Tehran, 19697 Iran; Department of Electrical and Computer Engineering, Bonab University, Bonab, East Azerbaijan Iran; Department of Clinical Neurological Sciences, Western University, London, ON Canada; Canadian Surgical Technologies & Advanced Robotics, Department of Electrical and Computer Engineering, Western University, London, ON Canada; Department of Surgery, Western University, London, ON Canada; Lawson Health Research Institute, And the Department of Clinical Neurological Sciences, Western University, London, ON Canada

**Keywords:** Writer’s cramp, Focal hand dystonia, Kinetic assessment, kinematic assessment, Abnormal motor movements

## Abstract

**Background:**

The assessment and treatment of writer’s cramp is complicated due to the variations in the forces and angles of involved joints. Additionally, in some cases compensatory movements for cramp relief further complicates assessment. Currently these variables are subjectively measured with clinical scales and visual assessments. This subjectivity makes it difficult to successfully administer interventions such as Botulinum toxin injection or orthotics resulting in poor efficacy and significant side effects.

**Method:**

A multi-sensor system was used to record finger and wrist forces along with deviation angles at the wrist, elbow and shoulder while 9 patients with writer's cramp performed a series of standardized tasks on surfaces inclined at different angles. Clinical, kinetic, and kinematic information regarding cramping was collected.

**Results:**

First, four tasks appeared to best predict cramp occurrence. Second, unique biomechanical profiles emerged for patients regarding force, angles and cramp severity. Third, cluster analyses using these features showed a clear separation of patients into two severity classes. Finally, a relationship between severity and kinetic-kinematic information suggested that primary cramping versus compensatory movements could be potentially inferred.

**Conclusions:**

The results demonstrate that using a set of standardized tasks and objective measures, individual profiles for arm movements and applied forces associated with writer’s cramp can be generated. The clinician can then accurately target the biomechanics specifically, whether it is with injection or other rehabilitative measures, fulfilling an important unmet need in the treatment of writer’s cramp.

**Electronic supplementary material:**

The online version of this article (doi:10.1186/s12984-016-0122-0) contains supplementary material, which is available to authorized users.

## Background

Focal hand dystonia is a movement disorder associated with atypical posturing of the upper limb during performance of task-specific, repetitive, or fine motor movements [1]. Writer’s cramp is a task-specific focal hand dystonia that negatively impacts handwriting ability. Patients may experience a range of symptoms, from muscular hyperactivity to increased grip force [2].

While physical rehabilitation strategies have been investigated in the literature [1, 3], most currently prescribed therapies are medical in nature [4–6]. Studies have reported on the use of a variety of treatments, including local injections of botulinum neurotoxin [4, 7, 8], prescription medication [5], transcranial magnetic stimulation [9] and deep brain stimulation [10]. Psychological counseling, relaxation exercises, and biomechanical training are often recommended as complementary rehabilitation strategies. Other than local injections of botulinum neurotoxin, none of the above mentioned therapies have been particularly popular or effective.

The first limitation comes from an inadequate motor characterization of the kinematic abnormalities of writer’s cramp patients. The involvement of multiple joints, in combination with the presence of compensatory movements makes it difficult to assess this disorder visually. Nevertheless, current standardized assessment tools such as the Arm Dystonia Disability Scale (ADDS) and the Writer’s Cramp Rating Scale (WCRS) rely solely on visual assessment by a clinician [11, 12]. 

Recent research on writer’s cramp has focused on quantifying individual motor abnormalities. For instance, Hermsdörfer et al*.* [2] used pen-related force and kinematic parameters to show that finger grip forces are important descriptors of individual impairment characteristics. Schneider et al*.* [13] showed similar deficits in pen kinematics and force parameters during writing. Yu et al*.* [14] suggested that writing difficulties may be associated with inaccurate in-air trajectory length and pathological wrist joint extension. To further investigate force and joint angle dysfunction, researchers have introduced and analyzed writing tasks of different complexities [13, 15, 16]. For instance, Zeuner et al. [11] implemented superimposed circle-drawing tasks and found that such tasks may increase sensitivity for detecting writer’s cramp.

While various devices have been used to measure discrete aspects of writer’s cramp, comprehensive, full-limb kinematic and kinetic measurements of writer’s cramp have not yet been implemented. The kinematic complexity and multi-segmental nature of this disorder makes it likely that each patient would have their own profile while sharing some similar motor abnormalities [11]. Therefore, several biomechanical parameters, including angular displacement at multiple joints, forces produced on the pen and the table, and secondary compensatory movements that make up the profile of writer’s cramp need to be assessed together to allow for effective treatment.

The second limitation is the poorly understood pathophysiology of the disorder. Studies using fMRI [17], transcranial magnetic stimulation [18], and kinematic assessment have implied that writer’s cramp patients may experience improper sensorimotor integration [2, 11, 13]. However limitations of the work to date exist both at the motor output (visible cramp) and the sensory input stage. Sensory inputs such as the size of the pen or visual input during writing can make a difference in the appearance of cramping symptoms.

Inaccurate proprioceptive input into the brain from the periphery may invoke abnormal motor responses [19]. While manipulating the writing conditions may prove a viable rehabilitation method in the future, current approaches for such manipulation to treat writer’s cramp are not well developed [6, 19]. Indeed it may be the variability in the sensory inputs that leads different motor output including the cramping (primary) and compensatory (secondary) movements.

This study was designed to answer some of these unmet needs. The first goal was to comprehensively define the kinematic and kinetic characteristics of writer’s cramp at multiple joints, across forces and angles at these joints. The second goal was to study the relationship between changes in the angles of the writing surface and the kinematics of cramping. Using these individual characteristics, the third goal was to kinematically characterize and define groupings across patients. This multiple feature-based clustering could potentially aid in the determination of primary dystonic versus secondary compensatory movements within writer’s cramp that could help the clinician in determining how to effectively apply interventions such as botulinum toxin and physical rehabilitation, which require specific and accurate targeting of cramp-involved muscles.

## Method

### Subjects

Nine writer’s cramp patients (3 females, age mean 56.7 (SD 2.4) years, 6 males, age mean 61.5 (SD 7.7) years) were recruited (Table 1). All participants gave written informed consent. The study was approved by the local Health Sciences Research Ethics Board (HSREB# 18643). None of the participants were receiving any form of treatment for their disorder.

**Table 1:** Demographics and clinical scale scores

Patient number	Gender	Age (years)	Affected upper limb	Symptom duration (years)	Beck anxiety inventory	Writer’s cramp rating scale	Arm dystonia disability scale	Unified dystonia rating scale
1	M	52	R	3	17	9	10	6
2	M	70	R	11	6	---^*^	4	4
3	F	55	R	7	5	2	6	4.5
4	M	70	R	15	7	6	5	6
5	M	58	R	45	8	5	4	4
6	M	55	L	7	22	7	5	5
7	F	55	R	2	23	9	8	6
8	M	64	L	3	11	---^a^	6	---^a^
9	F	60	R	3	32	---^a^	3	4.5

^a^data not available

### Setup

**Fig. 1:**
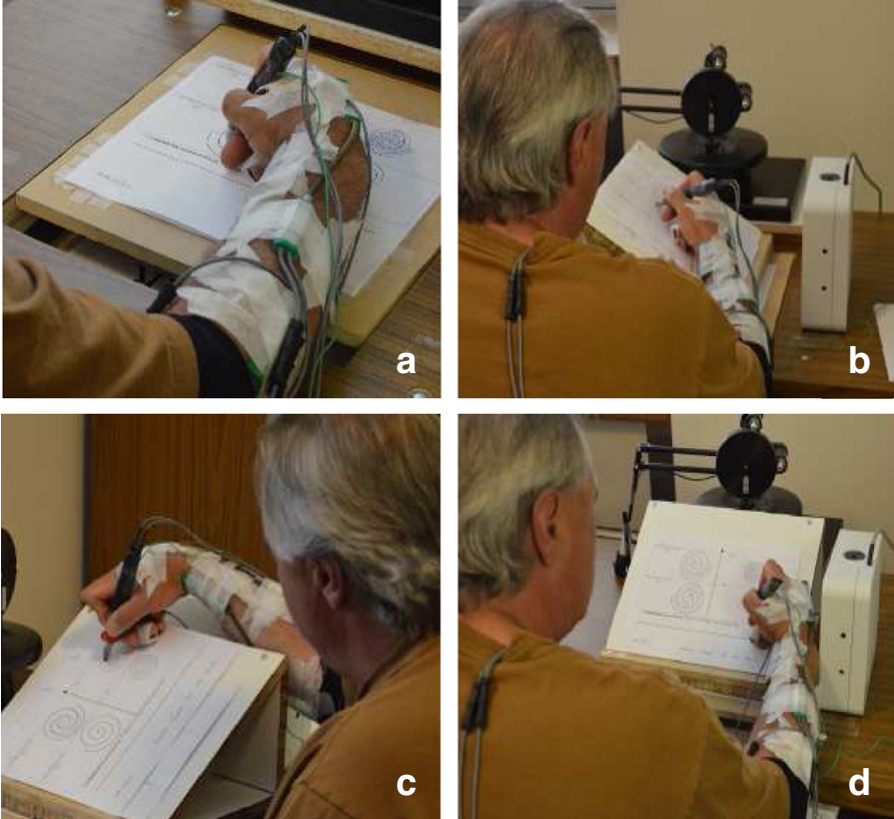
Experimental setup showing the kinematic sensors as well as **a** the flat (FLT), and the slanted surfaces: **b** towards the dominant hand (TDH), **c** towards the non-dominant hand (TNH), and **d** towards the participant (TPT)

Figure 1 shows the experimental setup. This setup was used in our previous work [20] to investigate the outcome of personalized rehabilitation strategies for patients with writer’s cramp. The setup includes a variety of force and joint angle sensors as well as a slanted surface, rotated to create three different inclined writing surfaces.

### Sensors

Kinematic assessment was carried out using surface attachment of three electro-goniometers and one torsiometer (NexGen Ergonomics Inc., Pointe Claire, Quebec). A twin axis goniometer (SG65) was placed on the wrist to measure wrist flexion/extension and radial/ulnar deviation. A single axis torsiometer (Q110) was placed on the forearm to measure wrist pronation/supination. A single axis goniometer (SG110) was placed on the elbow to measure elbow flexion/extension. A twin axis goniometer (SG75) was placed on the shoulder to measure shoulder flexion/extension and abduction/adduction. The position and orientation of the sensors were selected following the guidelines provided by the manufacturer and the calibration of the sensors was performed following the work presented in [21].

To measure grip force produced by thumb and index finger (i.e., thumb and index normal forces) during writing, the pen was equipped with two FlexiForce® sensors (NexGen Ergonomics Inc., Pointe Claire, Quebec). The pen was custom-made for this study. The writing surface was also pressure-sensitive using a force transducer (ATI Industrial Automation, Inc., Apex, California) to measure the normal hand force exerted during writing. Participants performed a series of standard scripted writing and drawing tasks while wearing the kinematic sensors. All sensors were non-invasive. The electro-goniometers directly provide joint angles and the force sensors directly provide thumb, index and hand forces during task completion.

### Surfaces

To evaluate the effect of manipulating the writing conditions on cramping, all tasks were performed on a horizontal surface as well as on three tilted surfaces. Participants completed writing and drawing tasks on the following surfaces: (1) flat (FLT), (2) tilted 30° towards the dominant hand (TDH), (3) tilted 30° towards the non-dominant hand (TNH), and (4) tilted 30° towards the participant (TPT).

The same set of tasks was performed in the same order on all surfaces. The order of surface presentation was randomized to minimize the effect of fatigue on the results. The writing surfaces were chosen as the method for this manipulation as it was envisioned that if a particular slant showed an improvement for reducing their particular cramp, then this could be used directly by the patient.

### Tasks

Participants performed a set of ten standardized tasks (summarized and numbered in Table 2). Tasks were chosen to provide the sensors with the most comprehensive information, allowing for accurate characterization of motor abnormalities. Holding the pen motionless above the paper (Table 2 - “hover”) provided a baseline for cramping occurring in the absence of volitional movement. Writing of a standard sentence (Table 2 - “sentence”) was chosen as cramps that occurred during this task were most representative of the original symptom.

**Table 2:** Scripted tasks: number, description and name

Task #	Task description	Task name
1	Hovering the pen over a fixed dot for 30 s	(Hover)
2	Spiral drawing (1): large, counter-clockwise	(Spiral I)
3	Spiral drawing (2): small, counter-clockwise	(Spiral II)
4	Spiral drawing (3): large, clockwise	(Spiral III)
5	Spiral drawing (4): small, clockwise	(Spiral IV)
6	Writing a standard sentence: “Today is a bright and sunny day”	(Sentence)
7	Sinusoid tracing left to right (1): low frequency, high amplitude	(Sine I)
8	Sinusoid tracing left to right (2): high frequency, high amplitude	(Sine II)
9	Sinusoid tracing left to right (3): low frequency, low amplitude	(Sine III)
10	Sinusoid tracing left to right (4): high frequency, low amplitude	(Sine IV)

Standardized drawing tasks break down the movements made during writing into simpler components. Kinematic assessment of these components allows for collection of detailed information about individual motor abnormalities. Hand movements localized to the wrist can be effectively measured with spiral drawing (Table 2 - “spiral I-IV”), as this task minimizes elbow and shoulder involvement. The more complex task of sinusoid tracing (Table 2 - “sine I-IV”), provides information about full arm motions, as they require elbow and shoulder involvement.

### Procedure

Each patient attended one session lasting approximately two hours in total (to complete the clinical scales and perform the assessment). Participants first completed the following clinical scales: the Beck Anxiety Inventory (BAI), the Unified Dystonia Rating Scale (UDRS), the Arm Dystonia Disability Scale (ADDS), and the Writer’s Cramp Rating Scale (WCRS); clinical scale scores are presented in Table 1. Scales were administered by trained research personnel.

After completing the scales, the kinematic sensors were attached to the affected arm using 3 M hypoallergenic micropore paper tape and the sensors were calibrated. Participants were seated on a height-adjustable chair in front of a desk. All tasks were completed on sheets fixed on a pressure-sensitive writing pad using a pen equipped with force sensors.

During each task, participants were asked to self-report when cramping began by using a signal word of their choosing (e.g. “cramp” or “now”). The time was recorded using an on-screen timer. This time was used to compare values from before the occurrence of the cramp to during cramping. After each individual task, participants were asked to rate the level of cramp intensity on a numerical scale from 0 being “no cramp” to 4 being “maximal discomfort” (considering level 2 as their usual level of cramping). Due to differences in individual symptomatology, “cramping” was defined as “the sensation you experience that interferes with your ability to write normally.”

Kinematic assessment began with a pre-adaptation task of writing a standard sentence on a flat surface. Participants then completed the group of tasks outlined in Table 2. The presentation order of the surfaces was randomized for each participant.

### Statistical and data analysis

Nine kinetic-kinematic measures were used to characterize distinct aspects of the tasks:

1. Thumb Force
2. Index Force
3. Hand Force
4. Wrist Flexion-Extension
5. Wrist Ulnar-Radial Deviation
6. Wrist Pronation-Supination
7. Elbow Flexion-Extension
8. Shoulder Flexion-Extension
9. Shoulder Abduction-Adduction

Statistical analyses were carried out with SPSS software, version 19.0 (SPSS Inc., Chicago, Illinois). The differences between the surfaces and the tasks were determined with a two-way analysis of variance (ANOVA) for repeated measures. Within-subject factors were ‘inclined surfaces’ and ‘standard tasks’. The level of significance was adjusted as *P* < 0.05.

In order to group the patients, two clustering analyses were performed. First, patients were grouped based on the applied forces, joint angles, and self-reported cramp severities. Second, to better understand how all the variables contribute to the overall picture of writer’s cramp, a different clustering approach, the Z-scores of the forces, angles, and cramp severity values of all patients on all surfaces are calculated. The Z-score is calculated based on the average and standard deviation of each parameter on a specific surface.

For instance, to calculate the Z-score of the Index force for patient#1 on the flat surface, the average and standard deviation of all Index forces on the flat surface are calculated and the Z-score is

1 $$ {Z^{P1}}_{In-FLT}=\frac{\left({F^{P1}}_{In-FLT}-{\overline{F}}_{In-FLT}\right)}{\sigma_{In-FLT}} $$

where Z^P1^_In − FLT_ is the Z-score of the Index force for patient 1 on the flat surface, F^P1^_In − FLT_ is the actual value of the Index force for patient 1 on the flat surface, ${\overline{\mathrm{F}}}_{\mathrm{In}-\mathrm{F}\mathrm{L}\mathrm{T}}$ is the average of all Index forces on the flat surface and σ_In − FLT_ is the standard deviation of all Index forces on the flat surface.

## Results

All nine participants completed the tasks on FLT and TDH surfaces. Eight participants performed the tasks on TNH, and only seven on TPT, due to technical issues. First, to ensure that task completion time did not greatly affect the results obtained for each task, the correlation between the task completion time and the cramp severity was investigated. There was no significant relationship between task completion time and the reported cramp severities (*r* = 0.34).

The relationship between normalized cramp latency and severity over all tasks and surfaces was also investigated. The normalized latency is defined as the division of the time it takes for the patient to report cramping (cramp latency) by the task completion time. Although not highly correlated (*r* = -0.62), there was a negative trend seen between normalized cramp latency and cramp severity. This indicates that more severe cramping events occurred sooner during the writing process.

**Fig. 2:**
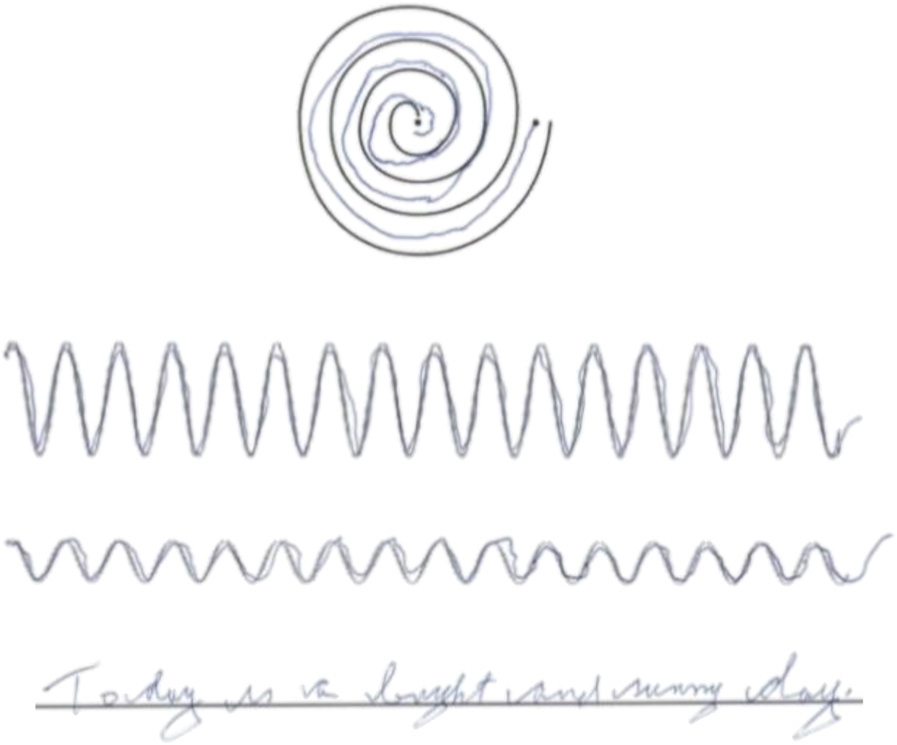
The selected tasks, (from top to bottom): Spiral II, Sine II, Sine IV, Sentence

Among all tasks completed by participants, four tasks in particular (Table 2 – Tasks# 3, 6, 8, and 10) presented the most severe level of cramping and were selected for further analysis. This selection was made based on averaging all self-reported cramp severities for each task performed on all surfaces. A sample of the selected tasks is illustrated in Fig. 2.

**Fig. 3:**
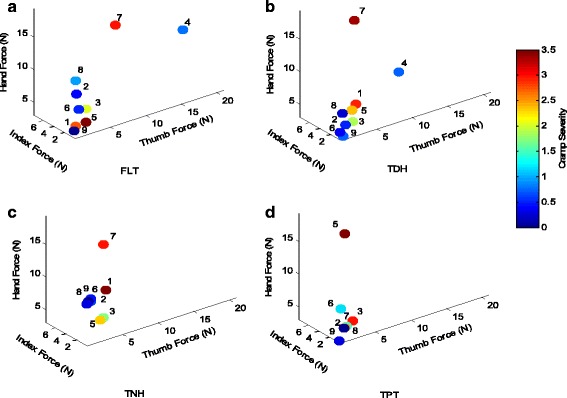
Mean values of the individual index finger, thumb, and hand forces of the participating patients with writer’s cramp on **a** FLT, **b** TDH, **c** TNH, and **d** TPT surfaces. Each point corresponds to an individual patient with the patient’s number next to it. The color of each point indicates the averaged cramp severity over the four selected tasks

**Fig. 4:**
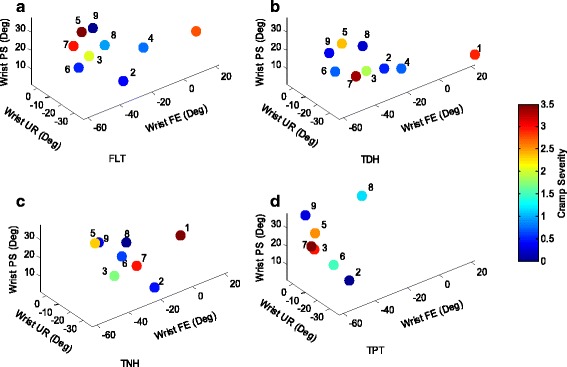
Mean values of the individual wrist flexion, ulnar deviation, and pronation of the participating patients with writer’s cramp on **a** FLT, **b** TDH, **c** TNH, and **d** TPT surfaces. Each point corresponds to an individual patient with the patient’s number next to it. The color of each point indicates the averaged cramp severity

**Fig. 5:**
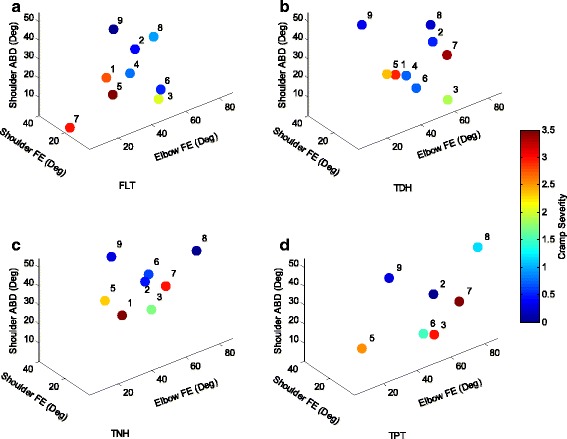
Mean values of the individual elbow flexion as well as shoulder flexion and abduction of the participating patients with writer’s cramp on **a** FLT, **b** TDH, **c** TNH, and **d** TPT surfaces. Each point corresponds to an individual patient with the patient’s number next to it. The color of each point indicates the averaged cramp severity

The first objective measures included forces applied on the pen and on the writing surface. Since the posture of the patient can affect the levels of the individual forces, all participants were positioned similarly. These forces were examined for the selected tasks performed on each of the surfaces. Figure 3 presents force measurements averaged over the selected tasks for the overall writing/drawing tasks. In this plot and all the following plots each point corresponds to an individual patient with the patient’s number adjacent to it and the color of each point indicates the average cramp severity level. Thumb force (mean 2.63 (SD 3.95) N) was generally higher than index force (mean 1.26 (SD 1.4) N) and patients applied higher hand forces (mean 7.83 (SD 4.3) N), than finger force. Compared to other surfaces, the TDH surface resulted in lower cramp severities (mean 1.4 (SD 1.2), *p* = 0.24). The second and third objective measures included joint angle measures at the lower and upper arm. Figure 4 presents the results of lower arm kinematic assessment (wrist angles) for each participant. In this figure the positive values correspond to wrist extension (X-axis), wrist ulnar deviation (Y-axis), and wrist pronation (z-axis). Fig. 5 presents the results of kinematic assessment for the upper arm. Positive values correspond to elbow flexion (X-axis), shoulder flexion (Y-axis), and shoulder abduction (Z-axis).

- Patient#1, who applied low forces during task completion, reported high cramp severities and showed the highest wrist extension. While the wrist flexion varied from -55° to -10° for most patients, for this patient it ranged from +16° to +32°. Change of radial deviation for this patient is also significantly different than others (comparing FLT and TDH surfaces: F(1,1) = 19.39, *p* = .015).
- Patient#2 generally applied low hand and fingers forces and reported low cramp severities on all surfaces. This patient showed very high wrist radial deviation on TNH and TPT surfaces.
- Patient#3 reported cramps close to the level of 2 on all surfaces except for TPT on which the level of cramping was higher. The applied forces were close to average. This patient had the lowest shoulder flexion and shoulder abduction and high elbow flexion on all surfaces.
- Patient#4 generally experienced low level of cramping while his thumb and index forces were high (reached 21.03 N, and 7.63 N on the FLT surface, respectively). The patient also showed low wrist flexion (-15.07° and -19.28° on FLT and TDH surfaces).
- Patients#5 and 6 had similar results regarding the forces and angles, however patient#5 generally experienced higher cramp severities on all surfaces. Patient#5 also presented higher wrist pronation on all surfaces while Patient # 6 had higher elbow flexion on all surfaces.
- Patient#7 reported the highest level of cramping (mean 3.1 (SD 0.2)) on all surfaces. The hand force for this patient was also the highest except for TPT. Elbow flexion seemed to be abnormal as the elbow was fully extended on FLT while elbow flexion reached 77.5° on TPT.
- For Patient#8 the level of cramp severity and the applied forces were low on all surfaces. The unique feature of this patient is that all angles seem consistent across the different surfaces.
- Patient#9 generally experienced the lowest cramp severities. For this patient the applied forces were also low on all surfaces.

**Fig. 6:**
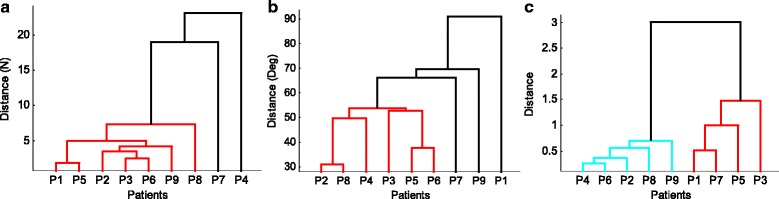
The dendrograms visualizing the hierarchy of clusters based on **a** applied forces, **b** joint angles, and **c** self-reported cramp severities from the 9 writer’s cramp patients

In order to group the patients, clustering analysis was performed. Patients were grouped based on the applied forces, joint angles, and self-reported cramp severities. The hierarchy of clusters is visualized by dendrograms in Fig. 6, using the data from the nine patients. Since not all patients were able to complete the tasks on TNH and TPT surfaces, the analysis was performed based on the data collected on the FLT and the TDH surfaces. The vertical axis on each dendrogram plot gives the multivariate distance between two patients or clusters of patients presented along the horizontal axis. The possible clusters are shown with different colors and multivariate distance is calculated using the Euclidean method.

To identify the intermediate clusters in Fig. 6, the horizontal lines connecting two clusters lie at a height where two or more clusters merge into one. Considering the applied forces and the measured joint angles, the patients may be clustered into a few nontrivial clusters. In Fig. 6(a) for distances < 5 N, clusters of {1,5} and {2, 3, 6, 9} can be identified. Similarly in Fig. 6(b), for distances < 55 degrees, there exist clusters of patients {2, 4, 8} and {3, 5, 6}. Finally, in Fig. 6(c) for distances < 1.5 N, the patients can be categorized into two major clusters, consisting of the patients {1, 3, 5, 7}, and the patients {2, 4, 6, 8, 9}.

The profiles of the Z-scores of the applied forces (thumb, index, and hand forces), the measured joint angles (wrist, elbow, and shoulder), and the cramp severities on all the surfaces for each individual patient may be found in the online Additional file 1. Positive angle Z-scores correspond to increased wrist flexion, wrist radial deviation, wrist pronation, elbow flexion, shoulder flexion, and shoulder abduction, respectively. The force, angle, and severity Z-scores for each patient are averaged to form an indicator for each patient.

**Fig. 7:**
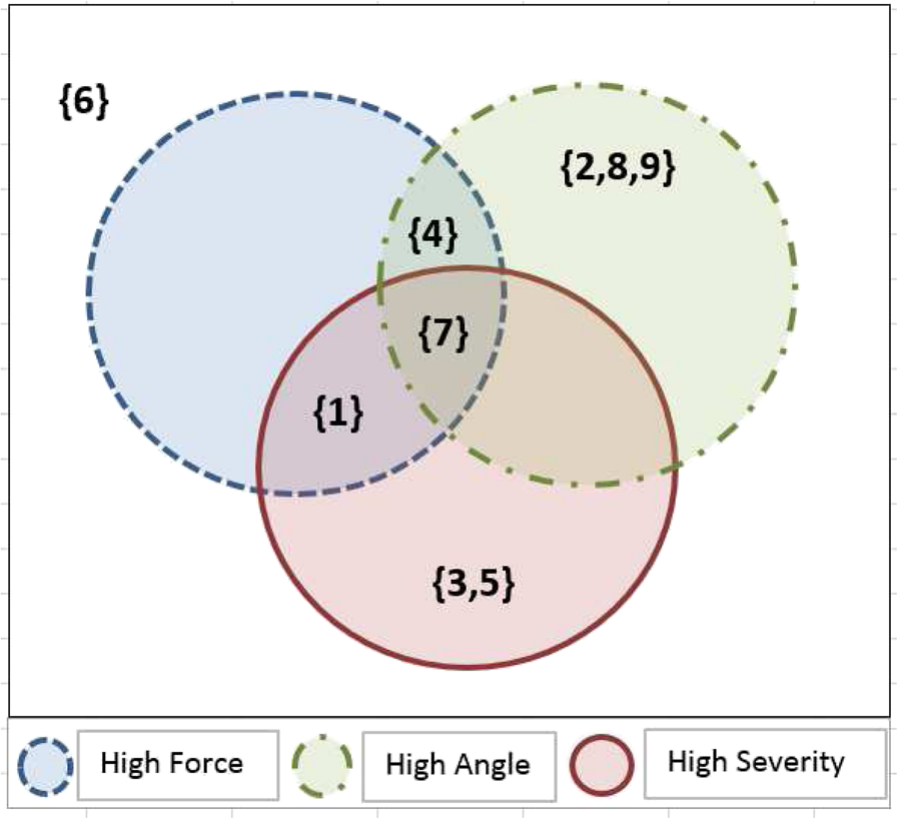
The Venn diagram, displaying the distribution of high force, high angle, and high severity in the 9 patients. Brackets indicate the patient IDs

The final indicators for each patient are summarized in Table 3. Each positive Z-score is considered as “higher than average force”, “higher than average angle”, and “higher than average severity”, for the applied forces, the joint angles, and the cramp severities, respectively. Figure 7 presents a Venn diagram, displaying the distribution of high force, high angle, and high severity in the 9 patients. The circles represent the patients with each of the characteristics and the numbers in the brackets indicate the patient IDs.

**Table 3:** The indicators of each patients’ force, angle, and cramp severity. The positive averaged Z-scores are considered as high indicators

Patient ID	Average force Z-scores	Average angle Z-scores	Average severity Z-scores
P#1	*0.30*	−0.56	*1.30*
P#2	−0.44	*0.13*	−1.00
P#3	−0.27	−0.12	*0.49*
P#4	*1.63*	*0.31*	−0.63
P#5	0.03	−0.10	*0.94*
P#6	−0.38	−0.41	−0.59
P#7	*1.40*	*0.29*	*1.38*
P#8	−0.61	*0.18*	−0.80
P#9	−0.78	*0.29*	−1.08

## Discussion

Using kinematic technology in combination with a variety of tasks and writing surfaces, we showed that individual patients exhibit a unique profile for their own version of writer’s cramp which can be measured and quantified. We were able to identify which tasks are most predictive of cramp occurrence. These tasks can be used by clinicians and are easily implementable. The patients were also classified into separate groups that could potentially help to indicate those patients whose kinematics were primary to the cramp versus those that were secondary.

The change in the writing surface inclination was considered as a manipulation to change the writing conditions. Since such changes also affect the posture of the upper extremity, we first presented the actual values for joint angles and applied forces (rather than the normalized values) on all surfaces. A classical classification was performed to observe if the patients necessarily experience the same kinematic and kinetic changes while writing and drawing on different inclined surfaces. Then the values were normalized using Z-score transformation to compare the performance of the patients with respect to the mean values on each surface.

Changing the inclination of the writing surface can affect the applied forces and the way they change when cramping occurs. One possible explanation could be that depending on the individual’s habit of writing, a change in the inclination results in either facilitating or delaying the cramp. Therefore in some patients these characteristics may be primary while in other patients they may be compensatory.

The relationships between severity, forces and angles across all surfaces are therefore clearly unique to each patient. The Z-Score plots and the Venn diagram plots (Fig. 7) for every patient in the severity, force, and angle domains emphasize the importance of categorization of the patients in order to understand the uniqueness of writer’s cramp.

An important issue with dystonia is that of primary abnormal movements due to cramping versus secondary compensatory movements that are voluntarily made by the patient to overcome the dystonic symptoms. The authors are aware of the fact that the presented method may not necessarily address this complex issue, but the clustering techniques discussed above may assist in further resolving it. The two clustering methods applied were able to separate the same patients based upon cramp severity, namely patients 1, 3, 5 and 7. In these patients where the severity Z-Score is negative, one can infer that the patient is experiencing less cramping. The characteristics of forces and angles can then be considered as those that have helped relieve the cramping. This then implies that the kinematic characteristics measured may be compensatory and that the cramping itself may be occurring in the opposite direction, potentially by activation of opposing muscles. By comparison, the force and angle characteristics of patients with above average cramping may be indicators of the cramping itself. In such cases, the kinematics would indicate the very joints and the associated muscles as being primarily involved in the cramp.

Finally, the authors are aware of the limitations in the process of data collection. First, some variability is always present in task design and we tried to minimize them as much as possible. For instance, most tasks (including sinusoid tracing and writing the sentence) are spread naturally from right to left and there is no way to avoid it. The distribution of spirals was also on either left or right side of the paper that could slightly affect the upper limb’s posture. Second, including control data could elaborate the interpretation of the results of this study. As a future work, one group of controls (a matching sample of healthy participants) could be considered to help understand how the proposed results represent abnormal values specific to writer’s cramp. Another control option could be patients with writing impairment due to non-neurological disorders, such as carpal tunnel. Third, in an ideal case, it would be unbiased if the assessment of cramp severity is performed based on a fully-objective and quantitative measure.

## Conclusions

Using a set of force and joint angle sensors, this research demonstrates that patients with writer’s cramp have unique kinematic profiles with interdependency between cramping and compensatory movements. These features may be brought out in individual patients with changing of the writing surfaces and with specific tasks. Such complex interdependencies cannot be identified by simple visual inspection of the patient’s writing. Once assessed together, such a profile for a patient could be a signature that would guide interventions such as local injection of Botulinum toxin or rehabilitation using an inclined plane or a device that specifically changes forces but not angles. This is the first time that such a comprehensive approach to understanding the biomechanics of writer’s cramp while cramping has been presented.

## Additional file

Additional file 1:
**Profiles of the** **Z-scores** **of the applied forces (Thumb, Index, and Hand), the measured joint angles (wrist, elbow, and shoulder), and the cramp severities on all surfaces for each individual patient.** (PDF 241 kb)

## Abbreviations

ADDS: arm dystonia disability scale
ADDS: arm dystonia disability scale
BAI: Beck anxiety inventory
FLT: flat surface
HSREB: health sciences research ethics board
SD: standard deviation
TDH: towards the dominant hand
TNH: towards the non-dominant hand
TPT: towards the participant
UDRS: unified dystonia rating scale
WCRS: writer’s cramp rating scale
WCRS: writer’s cramp rating scale
