# Supplementary figures and images for: Kinematic and kinetic assessment of upper limb movements in patients with writer's cramp
Source: J Neuroeng Rehabil. 2016 Feb 18;13:15. doi: 10.1186/s12984-016-0122-0 (PMC4759959; doi:10.1186/s12984-016-0122-0)

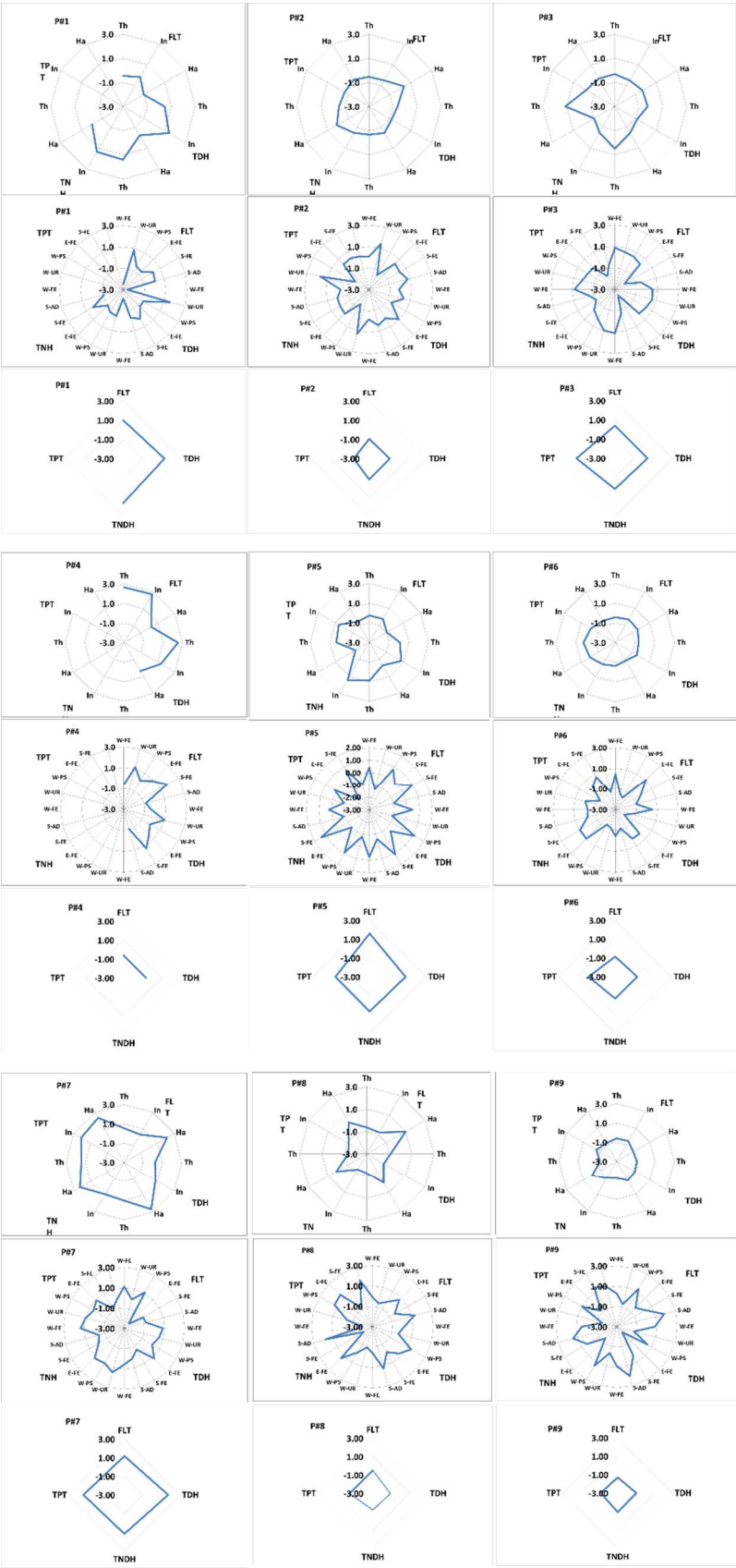

Supplement: Additional file 1: — Profiles of the Z-scores of the applied forces (Thumb, Index, and Hand), the measured joint angles (wrist, elbow, and shoulder), and the cramp severities on all surfaces for each individual patient. (PDF 241 kb) [file 12984_2016_122_MOESM1_ESM.pdf]
